# Low Cortisone as a Novel Predictor of the Low-Renin Phenotype

**DOI:** 10.1210/jendso/bvae051

**Published:** 2024-03-15

**Authors:** Alejandra Tapia-Castillo, Cristian A Carvajal, Jorge A Pérez, Alejandra Sandoval, Fidel Allende, Sandra Solari, Carlos E Fardella

**Affiliations:** Department of Endocrinology, School of Medicine, Pontificia Universidad Católica de Chile, Santiago 8330077, Chile; Centro Traslacional de Endocrinología UC (CETREN-UC), Santiago 8330033, Chile; Department of Endocrinology, School of Medicine, Pontificia Universidad Católica de Chile, Santiago 8330077, Chile; Centro Traslacional de Endocrinología UC (CETREN-UC), Santiago 8330033, Chile; Department of Endocrinology, School of Medicine, Pontificia Universidad Católica de Chile, Santiago 8330077, Chile; Centro Traslacional de Endocrinología UC (CETREN-UC), Santiago 8330033, Chile; Department of Endocrinology, School of Medicine, Pontificia Universidad Católica de Chile, Santiago 8330077, Chile; Centro Traslacional de Endocrinología UC (CETREN-UC), Santiago 8330033, Chile; Department of Clinical Laboratories, School of Medicine, Pontificia Universidad Católica de Chile, Santiago 7820436, Chile; Department of Clinical Laboratories, School of Medicine, Pontificia Universidad Católica de Chile, Santiago 7820436, Chile; Department of Endocrinology, School of Medicine, Pontificia Universidad Católica de Chile, Santiago 8330077, Chile; Centro Traslacional de Endocrinología UC (CETREN-UC), Santiago 8330033, Chile

**Keywords:** low-renin hypertension, cortisol, cortisone, aldosterone

## Abstract

A large proportion of patients with low-renin hypertension (LRH) correspond to primary aldosteronism (PA). However, some of these subjects have low to normal aldosterone. Since low renin is driven by excessive mineralocorticoids or glucocorticoids acting on mineralocorticoid receptors (MRs), we hypothesize that a low-cortisone condition, associated classically with 11βHSD2 deficiency, is a proxy of chronic MR activation by cortisol, which can also lead to low renin, elevated blood pressure, and renal and vascular alterations.

**Objective:**

To evaluate low cortisone as a predictor of low renin activity and its association with parameters of kidney and vascular damage.

**Methods:**

A cross-sectional study was carried out in 206 adult subjects. The subjects were classified according to low plasma renin activity (<1 ng/mL × hours) and low cortisone (<25th percentile).

**Results:**

Plasma renin activity was associated with aldosterone (r = 0.36; *P* < .001) and cortisone (r = 0.22; *P* = .001). A binary logistic regression analysis showed that serum cortisone per ug/dL increase predicted the low-renin phenotype (OR 0.4, 95% CI 0.21-0.78). The receiver operating characteristic curves for cortisone showed an area under the curve of 0.6 to discriminate subjects with low renin activity from controls. The low-cortisone subjects showed higher albuminuria and PAI-1 and lower sodium excretion. The association study also showed that urinary cortisone was correlated with blood pressure and serum potassium (*P* < .05).

**Conclusion:**

This is the first study showing that low cortisone is a predictor of a low-renin condition. Low cortisone also predicted surrogate markers of vascular and renal damage. Since the aldosterone to renin ratio is used in the screening of PA, low cortisone values should be considered additionally to avoid false positives in the aldosterone–renin ratio calculation.

One-third of patients with hypertension have low or suppressed renin, which suggests systemic volume expansion and mineralocorticoid receptor (MR) activation [1]. Low-renin hypertension (LRH) is a prevalent phenotype worldwide depending on age and race [2], with a higher prevalence in African Americans, around 45%, vs 32% in non-Hispanic White people [3-5]. Indeed, there are endocrine pathological conditions associated with LRH, such as primary aldosteronism (PA), the syndrome of apparent mineralocorticoid excess (AME), atypical forms of congenital adrenal hyperplasia, and alterations in the activity of the MR or epithelial sodium channel (Liddle syndrome).

A large proportion of LRH corresponds to patients with PA. PA is the most prevalent form of endocrine hypertension, with an estimated prevalence of 5% to 10% in the general hypertensive population, and it occurs due to unregulated MR activation by excessive circulating aldosterone, increasing renal sodium and water reabsorption [6, 7]. The prevalence of PA might be higher than currently reported, since a continuum of renin-independent aldosterone secretion shows an extended spectrum of PA from normotension to severe arterial hypertension [8]. Subclinical forms of PA are probably responsible for a large proportion of patients with the low-renin phenotype associated with elevated blood pressure (BP) or arterial hypertension. However, PA and milder form of PA only explain around the 15% of LRH [9]; hence, other conditions could explain the remaining cases of LRH with aldosterone-independent MR activation mechanisms, such as a glucocorticoid-mediated MR activation, as occurs in the AME [10].

AME is a rare genetic autosomal recessive disorder caused by the presence of a pathogenic variant in the HSD11B2 gene (NG_016549), causing a severe deficiency in 11β-hydroxysteroid dehydrogenase type 2 (11βHSD2) activity. Under normal conditions, the activation of the MR by cortisol does not occur because the enzyme 11βHSD2 efficiently inactivates cortisol to cortisone, avoiding the binding of cortisol to MR [11-13]. The clinical classic features are childhood-onset hypertension, hypokalemia, and alkalosis with low plasma renin. The partial 11βHSD2 activity deficiency, which has been called nonclassic AME (NCAME), is a mild form of classic AME, without the classic features of severe disease and with a prevalence around of the 7% in general population [14]. It is characterized by a normal or elevated BP, with a high serum cortisol to cortisone (F/E) ratio and concomitant low cortisone levels [14]. We previously showed that low serum cortisone has been reported to be one of the best predictors of MR activation in subjects with NCAME [14]. Since low renin is driven by excessive mineralocorticoids or glucocorticoids acting on the MR, then we hypothesize that a low-cortisone condition, associated classically with 11βHSD2 deficiency, is a proxy of chronic MR activation by cortisol, which also can lead to low renin, elevated BP, and renal/vascular alterations. Thus, the current aim of this study is to evaluate low cortisone levels as a predictor of low renin phenotype and its association with endothelial and renal damage, and proinflammatory activity.

## Materials and Methods

### Subjects

We performed a cross-sectional study in Chilean subjects from a primary care cohort of 402 individuals previously recruited from different centers in Santiago, Chile. The cohort included subjects of both sexes with similar socioeconomic status and ethnicity. The protocol was performed according to the principles of the Declaration of Helsinki and approved by the local ethics committee of the Faculty of Medicine, Pontificia Universidad Católica de Chile (certificate of approval CEC-MEDUC 14-268 [FONDECYT 1150437] and CEC-MEDUC 12-207 [FONDECYT 1130427]).

**Figure 1.**
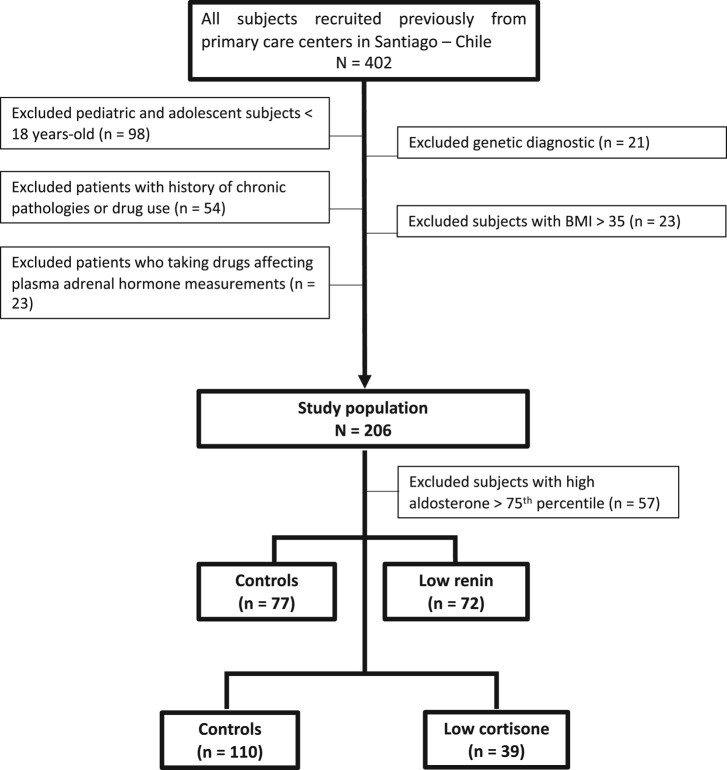
Study design and selection of subjects. Flow chart showing the study design, exclusion criteria and categorization of subjects. We excluded subjects with high aldosterone levels (>75th percentile, aldosterone >11.35 ng/dL). The subjects were classified according to low plasma renin activity (<1 ng/mL × hours) (n = 77) and then subjects were classified by low cortisone with a serum cortisone lower than the first quartile (Q1) (<25th percentile; E < 1.98 µg/dL).

In this study, we included both normotensive and hypertensive subjects. All subjects had sodium diet ad libitum and declared that they did not ingest any herbal products or extreme diets during the month prior to the analysis. We excluded pediatric and adolescent subjects (<18 years of age), obese subjects (body mass index [BMI] > 35 kg/m^2^), and patients with a genetic diagnosis of familial hyperaldosteronism or a history of chronic pathologies, such as renal failure, heart failure, diabetes mellitus, chronic liver damage, or endocrinopathies. Also, we excluded subjects with high serum aldosterone levels (higher than the 75th percentile [11.35 ng/dL]) (Fig. 1). Patients treated with drugs affecting plasma adrenal hormone measurements, such as glucocorticoids (eg, cortisol or prednisone) or mineralocorticoids (eg, fludrocortisone), spironolactone (MR antagonist), oral contraceptives [15, 16], or who were using antihypertensive drugs that affect the renin–angiotensin–aldosterone system (RAAS), such as beta-blockers, angiotensin-converting enzyme inhibitors, angiotensin II receptor blockers, and diuretics, were also excluded from this study. Patients treated with the antihypertensive drugs previously described were started on amlodipine or doxazosin for at least 4 weeks before any determination.

After the exclusion criteria were applied, 149 subjects were included in this study (Fig. 1). The subjects were classified as having low renin with a low plasma renin activity (PRA) (<1 ng/mL × hours) according to previously described methods [17]. Similarly, subjects were classified as having low cortisone with a serum cortisone lower than the first quartile (Q1) (*P* < 25th percentile; cortisone ≤ 1.98 µg/dL). Quartiles were determined on the basis of all 149 studied subjects.

### Clinical Parameters

All subjects in this study had clinical records and physical examinations that included age, height, weight, BMI, and BP [18]. Briefly, 3 BP measurements were obtained from the right arm at consecutive 5-minute intervals using an oscillometric method (Dinamap CARESCAPE V100, GE Health Care, Medical Systems Information Technologies, Milwaukee, WI) in a seated position, according to published recommendations. Hypertension-AHA guidelines were followed to identify BP categories in our cohort [19].

### Biochemical Assays

A biochemical profile was performed: the fasting blood samples (08:00-10:00 Am) from every subject, in the sitting position with at least a 15 minutes of rest, were obtained to measure different biochemical parameters as levels of cortisol, cortisone, PRA, aldosterone, sodium, potassium, and creatinine. The samples were used immediately or were stored at −80 °C. Serum aldosterone and PRA were measured by radioimmunoassay using a commercial kit (Siemens Coat-A-Count Kit; Cat# TKAL2, RRID:AB_2737007. Los Angeles, CA, and DiaSorin, Cat# CA1533, RRID:AB_2736926. Stillwater, MN, respectively). Serum and urinary cortisol and cortisone were quantified using liquid chromatography combined with tandem mass spectrometry according to previously reported methods [14].

We measured the serum and urinary electrolytes sodium (Na^+^) and potassium (K^+^) using methods previously described [20]. We also calculated 24-hour sodium excretion (mEq/24 hours), fractional excretion of sodium in 24 hours (%), fractional excretion of potassium (FEK, % in 24 hours) and the Na^+^/K^+^ ratio. We also calculated the transtubular transport of potassium gradient (osmometer from Advanced Instruments 3300, Waters). Urinary creatinine was measured by the Jaffe method with automated equipment (Modular Analytics, Roche, Germany).

Albumin excretion was evaluated using 24-hour urine, which was measured by a turbidimetric immunoassay (Albumin; Roche, Germany) using a Hitachi Automatic Analyzer 7600 (Hitachi, Japan). Plasminogen activator inhibitor (PAI-1) activity was determined by enzyme-linked immunosorbent assay (ANIARA; catalog #RK019A, RRID:AB_3086772), and metalloproteinase 9 was determined by zymography as previously described [21]. High sensitivity C-reactive protein was evaluated by a nephelometric assay (BN ProSpec Systems, Siemens, USA). Urine creatinine was measured with a colorimetric assay (Roche, Indianapolis, IN) using a Hitachi Automatic Analyzer 7600 (Hitachi). Human lipocalin-2 (NGAL) was quantified by sandwich enzyme immunoassay using commercial reagents and standards according to the manufacturer's protocols (DLCN20, RRID:AB_2894833. R&D System, Inc., USA).

### Data Analysis

Categorical variables are shown as percentages. Differences in sex were analyzed by the chi-squared test. Normality was assessed using the Kolmogorov–Smirnov test. In situations in which a variable was not normally distributed, a bootstrapping procedure with 1000 iterations was performed. The variables are reported as medians and interquartile ranges (Q1-Q3). The comparisons were performed by Mann–Whitney analyses. The correlations were performed by the Pearson test with bootstrapping and adjusted linear regression for age and sex, and BMI was used to evaluate the association between aldosterone, cortisone, and biochemical variables. Receiver operating characteristic (ROC) analysis was used to discriminate the low-renin phenotype from control subjects. We also perform logistic regression analyses, using Wald selection, to find predictive variables, such as aldosterone, cortisol, cortisone, sex, BMI, age, BP (continuous variables) of low-renin phenotype (categorical variable). A *P* value of .05 was considered to be statistically significant. Analyses were performed using SPSS 20 and GraphPad Prism v9.0 software.

## Results

### Clinical and Biochemical Characteristics in Serum

We identified 72 out of 149 subjects with a low-renin phenotype (48%). The baseline characteristics of both groups are shown in Table 1. Subjects with low renin levels had higher age, systolic BP (SBP) and diastolic BP (DBP). We observed that subjects with low renin also had lower aldosterone (6.7, 3.6-9.7, vs 7.7, 5.5-8.9, ng/dL; *P* < .001) and cortisone (2.1, 1.8-2.3, vs 2.2, 2.0-2.6 µg/dL; *P* = .003) levels than the control subjects. Additionally, we observed increased urinary albumin excretion in the low-renin group vs the control group (4.4, 2.9-7.3, vs 2.6, 1.2-5.2, µg/mg creatinine; *P* = .005) (see Table 2).

**Table 1.** Clinical and serum biochemical characteristics of the studied subjects

Parameters	Controls	Low renin	*P* value
N	77	72	
Sex, male/female	45/32	20/52	.002
Age, years	41.3 (29.1-50.9)	46.8 (39.5-59.8)	<.001
BMI, kg/m^2^	27.8 (24.9-29.8)	27.5 (25.7-29.5)	.2
SBP, mmHg	119.5 (114.1-133.5)	136.3 (125.3-143.7)	.007
DBP, mmHg	79.2 (74.80-87.0)	82.3 (78.7-92.0)	.04
Aldosterone, ng/dL pmol/L	7.7 (5.45-8.9) 213.8 (151.4-247.2)	6.7 (3.6-9.7) 186.1 (100-269.4)	<.001
PRA, ng/mL×hour ng/L × second	1.7 (1.30-2.3) 0.47 (0.36-0.64)	0.4 (0.3-0.6) 0.1 (0.08-0.16)	<.001
ARR	4.2 (2.70-5.3)	13.4 (8.8-23.3)	<.001
Serum Na, mEq/L	140 (139-142)	141 (141-143)	.09
Serum K, mEq/L	4.2 (4.10-4.4)	4.2 (3.9-4.4)	.82
Hs-CRP, mg/L	1.4 (0.58-3.2)	1.2 (0.8-5.0)	.5
PAI-I, ng/mL	14.3 (9.77-17.4)	20.8 (11.7-28.2)	.1
Serum cortisol, µg/dL nmol/L	9.5 (7.14-12.9) 261.7 (196.7-355.4)	10.7 (8.9-13.5) 294.7 (245.2-371.9)	.1
Serum cortisone, µg/dL nmol/L	2.2 (2.0-2.6) 60.9 (55.4-72.0)	2.1 (1.8-2.3) 58.1 (49.8-63.7)	.003
Serum F/E ratio	4.1 (3.2-5.8)	4.6 (3.8-6.2)	.6
NGAL, ng/mL	113.3 (74.0-139.2)	139.6 (92.9-155.9)	.1

Values correspond to median (Q1-Q3). Mann–Whitney test.

Abbreviations: ARR, aldosterone to renin ratio; F/E, cortisol to cortisone ratio; hs-CRP, high-sensitivity C-reactive protein; K^+^, potassium; MMP, metalloproteinase; Na, sodium; NGAL, neutrophil gelatinase-associated lipocalin.; PAI-1, plasminogen activator inhibitor; PRA, plasma renin activity.

**Table 2.** Urinary biochemical characteristics of the studied subjects

	Controls	Low renin	*P* value
Urinary Na, mEq/24 hours	183.5 (125.3-231.3)	229.0 (100.0-267.0)	.3
FENA, % 24 hours	0.6 (0.5-0.8)	0.8 (0.6-0.9)	.3
Urinary K, mEq/24 hours	54.5 (43.0-69.0)	47.0 (37.0-62.0)	.4
FEK, % 24 hours	7.1 (5.4-8.7)	7.5 (5.8-8.7)	.1
Urinary Na/K ratio	3.3 (2.3-4.5)	3.6 (3.1-4.9)	.5
TTKG	4.3 (3.5-5.4)	5.3 (3.5-6.3)	.3
Glomerular filtration rate	109.8 (94.3-121.3)	95.3 (84.5-104.7)	.04*
Urinary albumin, µg/mg Crea	2.6 (1.2-5.2)	4.4 (2.9-7.3)	.005*
Free urinary cortisol, µg/g creatinine	10.1 (7.6-19.0)	10.8 (3.9-15.3)	.3
Free urinary cortisone, µg/g creatinine	29.1 (20.1-40.9)	27.9 (11.9-37.7)	.01*
Urinary F/E ratio	0.4 (0.3-0.5)	0.4 (0.3-0.5)	.6

Values correspond to median (Q1-Q3).

Abbreviations: FEK, fractional excretion of potassium; FENa, fractional excretion of sodium; K^+^, potassium; Na, sodium; TTKG, transtubular potassium gradient.

*P < .05, Mann–Whitney test.

### Prediction Regression Model and Correlation Studies

**Figure 2.**
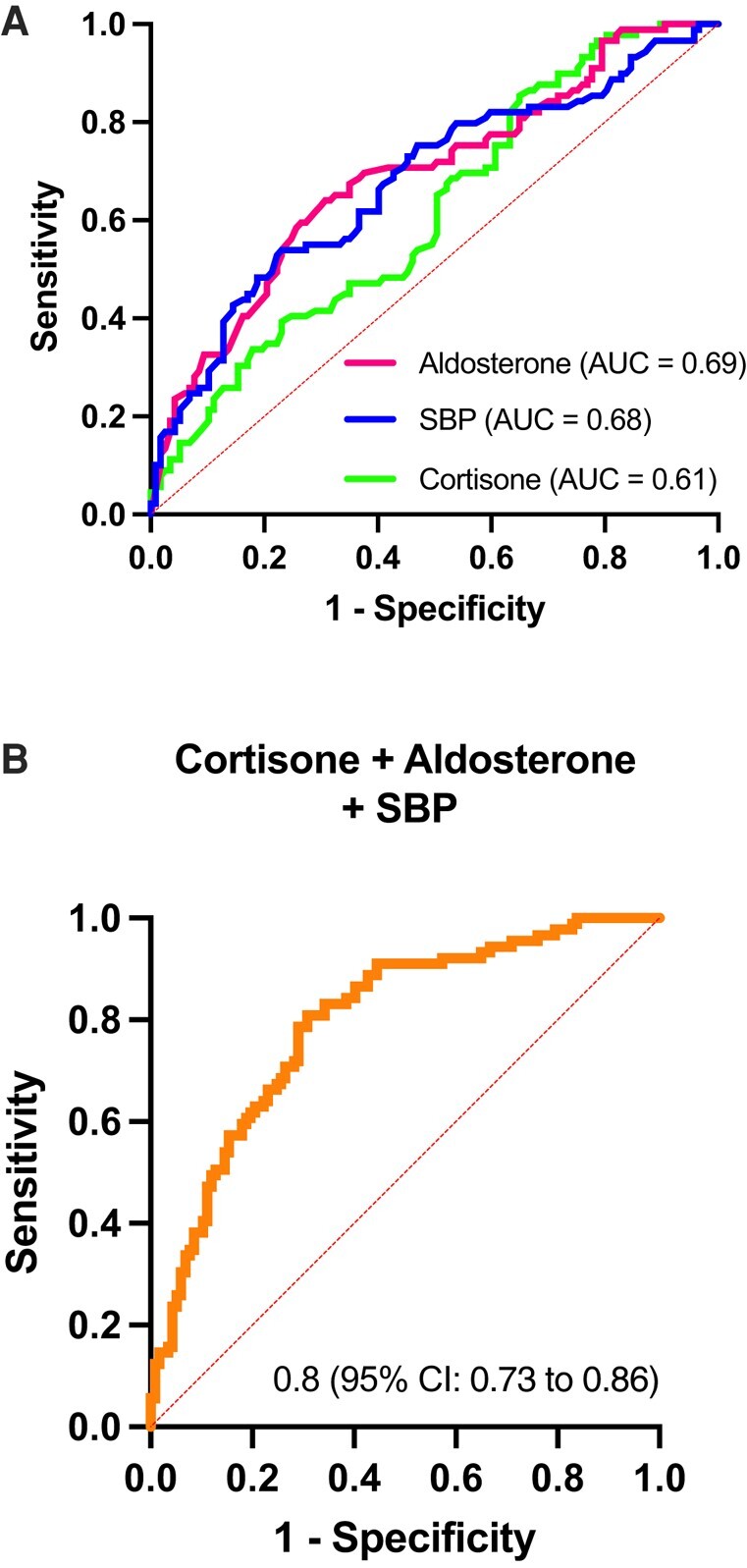
Logistic regression model and evaluation of diagnostic efficacy using ROC curve for low-renin phenotype and different biochemical variables. Aldosterone, cortisol, cortisone, sex, BMI, age, and blood pressure were considered to be continuous variables. (A) ROC curve for serum cortisone, plasma aldosterone, systolic blood pressure (SBP); (B) combination variable; cortisone + aldosterone + SBP.

We performed a logistic regression to determine the variables that predicted a low-renin phenotype in the total group. The significant features with *P* < .05 were aldosterone (OR 0.8, 95% CI 0.81-0.93, *P* = .001), cortisone (OR 0.4, 95% CI 0.21-0.78, *P* = .007) and SBP (OR 1.035, 95% CI 1.019-1.052, *P* = .001). To assess the discriminatory capacity of cortisone and aldosterone levels in the identification of the low-renin phenotype, we applied ROC curve analysis to the data, and the area under the curve (AUC) for serum cortisone was 0.62 (95% CI 0.5-0.69; *P* = .004) (Fig. 2A) and urinary cortisone 0.60 (95% CI 0.52-0.67; *P* = .02). We identified that serum cortisone levels of 2.7 µg/dL had a sensitivity of 85% and specificity of 35% to discriminate between subjects with low renin and subjects with normal renin, and that urinary cortisone levels of 43.1 µg/24 hours had a sensitivity of 70% and a specificity of 50% to discriminate between subjects with low renin and control subjects. Using a serum cortisone threshold value of 2.7 µg/dL, 86.5% of the subjects with low renin displayed low cortisone. In the same way, ROC analysis showed an AUC for serum aldosterone of 0.69 (95% CI 0.62-0.76; *P* = .0001) (Fig. 2A). An aldosterone concentration of 7.35 ng/dL had the best sensitivity (64%) and specificity (70%) to discriminate between subjects with low renin and subjects with normal renin. Using an aldosterone threshold value of 7.35 µg/dL, 35.9% of the subjects with low renin displayed high aldosterone. We also performed ROC curve analyses for combined variables to discriminate low renin levels from normal renin levels. Cortisone, aldosterone, and SBP had an AUC of 0.80 (95% CI 0.73-0.86, *P* < .0001) (Fig. 2B) in the identification of the low-renin phenotype.

We performed a comparison of the group with low and normal cortisone (both adjusted with aldosterone <75th percentile; 11.35 ng/dL) **(**Table 3). We observed that subjects with low serum cortisone levels had higher age (50.5, 41.7-56.4, vs 41.7, 31.4-52.2; *P* = .0004), BMI (28.5, 26.3-30.2, vs 27.1, 24.6-29.2; *P* = .03), urinary albumin (9.5, 6.1-21.4, vs 7.0, 4.3-11.4; *P* = .01), and PAI-1 (19.2, 11.9-24.1, vs 14.9, 9.2-24.8; *P* = .04), and lower urinary sodium excretion (126, 96.5-185, vs 162, 121-196; *P* = .04) than subjects with normal serum cortisone. These differences also persist with urinary cortisone levels. We did not observe statistical differences in the F/E ratio values: we only found that 27% of subjects had a high F/E in the low-cortisone group.

**Table 3.** Clinical and biochemical characteristics of subjects with low and normal cortisone

	Normal cortisone	Low cortisone	*P* value
N	110	39	
Gender, male/female	52/58	13/26	.2
Age, years	41.7 (33.0-52.5)	50.6 (44.0 -56.0)	.001*
BMI, kg/m^2^	27.1 (24.6 -29.2)	28.5 (27.1- 30.2)	.002*
SBP, mmHg	128.3 (116.3-139)	128.3 (119.2-146)	.1
DBP, mmHg	81.7 (75-89.3)	82.7 (77.5-92.5)	.3
Aldosterone, ng/dL pmol, L	6.7 (5.2-8.2) 186.1 (144.4-227.7)	6.1 (3.7-8.5) 169.4 (102.7-236.1)	.2
Plasma renin activity, ng/mL × hour ng/L × second	1.2 (0.6-2.0) 0.33 (0.16-0.55)	0.8 (0.5-1.4) 0.22 (0.14-0.38)	.04*
Serum Na, mEq/L	141 (140-142)	141 (139-142.5)	.9
Serum K, mEq/L	4.2 (4.0-4.5)	4.2 (4.0-4.47)	.6
Hs-CRP mg/L	1.4 (0.6-2.5)	1.2 (0.67- 2.9)	.7
glomerular filtration rate, MDRD-4	107.8 (92.2-121.6)	104.5 (93- 118.1)	.8
PAI-I, ng/mL	13.7 (9.2-24.8)	19.5 (14.5-23.9)	.01*
Urinary Na, mEq/24 hours	165 (123-197)	119 (89.5-190)	.01*
FENA, 24 hours %	0.7 (0.5-0.9)	0.7 (0.5-0.9)	.8
Urinary K, mEq/24 hours	51 (39-64)	46 (37-58)	.2
FEK, 24 hours %	7.1 (5.9-8.6)	7.3 (6.0-9.4)	.5
Urinary Na/K ratio	3.2 (2.2-4.1)	2.8 (1.9-4.7)	.5
Urinary albumin, mg/24 hours	7.0 (4.3-11.5)	10.3 (7.1-22.2)	.01*

Values correspond to median (Q1-Q3).

Subjects were classified having a low cortisone with a serum cortisone lower than the first quartile (Q1) (*P* < 25th; E ≤ 1.98 ug/dL) and normal cortisone greater than the 25th percentile. The subjects were adjusted by aldosterone <75th percentile; 11.35 ng/dL).

Abbreviations: DBP, diastolic blood pressure; FEK, fractional excretion of potassium; FENA, fractional excretion of sodium; hs-CRP, high sensitivity C reactive protein; K, potassium; K, potassium.; Na, sodium; Na, sodium; PAI-1, plasminogen activator inhibitor; SBP, systolic blood pressure.

*P < .05, Mann–Whitney test.

*A *P* < .05 is considered statistically significant.

We also investigated the correlation analysis adjusted by age, sex, and BMI in the total group. We observed that PRA was positively associated with serum cortisone (r = 0.31; *P* < .001), urinary cortisone (r = 0.15; *P* = .02), and aldosterone (r = 0.26; *P* < .001) (Table 4). SBP and DBP were correlated with both serum cortisone (r = −0.13; *P* = .03; r =−0.15; *P* = .02) and urinary cortisone (r = −0.23; *P* < .001; r = −0.2; *P* = .003), respectively. Moreover, the association study showed that serum cortisone was negatively correlated with urinary albumin (r = −0.13; *P* = .03) and PAI-1 (r = −0.12; *P* = .05), and urinary cortisone was correlated with serum sodium (r = −0.13; *P* = .04) and potassium (r = 0.17; *P* = .01) and urinary sodium (r = 0.2; *P* = .003) and potassium (r = 0.2; *P* = .002) (see Table 4).

**Table 4.** Correlation analysis between cortisone, aldosterone, and biochemical variables adjusted by sex and age

	Serum Cortisone		Urinary cortisone		Aldosterone	
	r	*P* value	r	*P* value	r	*P* value
SBP	−0.13	.038	−0.23	<.001	0.01	.9
DBP	−0.15	.02	−0.2	.003	0.02	.7
PRA	0.31	<.001	0.15	.019	0.26	<.001*
Serum Na mEq/L	0.03	.3	−0.13	.04	−0.13	.06
Serum K, mEq/L	0.03	.3	0.17	.01	−0.09	.22
PAI-I	−0.12	.05	−0.07	.15	0.08	.24
Glomerular filtration rate	−0.05	.23	−0.02	.4	−0.21	.003*
Urinary Na, mEq/24 hours	−0.004	.48	0.2	.003	−0.17	.014*
FENA	0.05	.2	0.05	.23	−0.12	.08
Urinary K	−0.08	.13	0.21	.002	−0.07	.13
FEK	−0.06	.19	0.05	.2	0.18	.01*
Urinary Na/K ratio	0.02	.38	−0.03	.3	−0.2	.003*
Urinary albumin	−0.13	.03	0.03	.3	0.1	.15

Bootstrapped Pearson correlation.

Abbreviations: BMI, body mass index; DBP, diastolic blood pressure; E, cortisone; F, cortisol; FEK, fractional excretion of potassium; FENA, fractional excretion of sodium; hs-CRP, high sensitivity C reactive protein; K, potassium; Na, sodium; PAI-1, plasminogen activator inhibitor; PRA, plasma renin activity; SBP, systolic blood pressure.

*A *P <* .05 is considered to be significant.

Additionally, aldosterone was correlated with urinary sodium (r = −0.17; *P* = .01), FEK (r = 0.18; *P* = .01), urinary Na/K (r = −0.2; *P* = .003), and glomerular filtration rate (r = −0.21; *P* = .003) (Table 4).

## Discussion

This is the first study showing low cortisone levels as a predictor of a low-renin phenotype. Furthermore, we found that cortisone was associated with high BP and biomarkers of vascular (PAI-1) and renal damage (albuminuria). We also found that aldosterone was associated with an impaired urinary electrolyte concentration and altered renal function (estimated glomerular filtration rate).

The results of our study showed that either low serum cortisone or urinary cortisone predicted a low-renin condition. The ROC analysis with cortisone can discriminate 60% of subjects with low renin (AUC 0.6), although it has a low predictive value, which is similar to serum aldosterone *per se*; however, the combination of both variables with the SBP can discriminate 80% of the subjects with low renin, suggesting other causes of low renin associated with glucocorticoid-mediated MR activation, such as classical AME and NCAME syndrome, which consequently decrease PRA and could modify the aldosterone–renin ratio, generating a false positive. However, there are other pathologies that can lead to a low-renin condition, as occurs in Liddle syndrome, which is the most common cause of LRH in the African American population. This is frequent due to mutations in *SCNN1A*, *SCNN1B*, and *SCNN1G*, allowing salt and water reabsorption independent of MR activation [22].

When analyzing subjects classified according to low cortisone, we found that 27% of them had a high F/E ratio, which suggests a partial deficiency of 11βHSD2. In this respect, it is proposed that the thresholds for confirmatory tests are relatively arbitrary and there is likely a continuum that exists below classical diagnostic thresholds. Thus, the threshold considered in this study was of serum F/E ≥ 6.0 (>75th percentile), higher than that published in the *Journal of Clinical Endocrinology and Metabolism* in 2018 (F/E ≥ 4.43) [14], and if we consider the previously published threshold, the percentage increases approximately to 50% of the population with NCAME. Another possibility is that the percentage of patients with NCAME could be underestimated in patients who had lost their circadian rhythm, altering morning cortisol levels and thus the F/E ratio. According to previous publications patients with low-renin essential hypertension have an increased F/E ratio both in urine and serum samples compared with normotensive subjects, which is negatively associated with serum aldosterone and PRA, as occurs in AME syndrome [14]. These findings are similar to those found in the Framingham study, which described subjects with LRH having a bimodal aldosterone distribution, suggesting 2 different pathophysiological phenotypes associated with MR activation [23]. One is associated with mineralocorticoid (ie, aldosterone) and the other with glucocorticoid (ie, cortisol or deoxycorticosterone) activation, as occurs in AME where cortisol metabolism is impaired [24].

On the other hand, in subjects with low cortisone and normal F/E ratio, NCAME cannot be considered. In these patients, the low cortisone is determined by a lower but normal cortisol. This situation is common in pathologies that have a wide phenotypic spectrum, with continuous variables, where the cut-off point is highly variable; for instance, as occurs in PA, various cut-off points are reported for the aldosterone to renin ratio. In this sense, the cut-off used in this study was very strict and therefore we observed a lower number of patients with high F/E. Moreover, the high serum F/E in NCAME could be observed for an increase in plasma cortisol half-life, and a decrease in circulating cortisone values is a better indicator of inhibition of 11βHSD2 in vivo [25-27]. Otherwise, we observed subjects with low cortisone and normal renin (8.7%), half of whom had a high F/E and a trend to higher FEK, compatible with a low-renin phenotype. We should expect a low-renin phenotype with a decrease in renin over time, associated with higher sodium intake or other environmental challenges affecting activity or expression of 11βHSD2.

This study showed that subjects with low cortisone are older than subjects with normal cortisone, suggesting a reduction in 11βHSD2 expression with age possibly due to the *HSD11B2* gene being susceptible to methylation because it has major CpG islands in the promoters, exon 1, and exon 5 [28-30]. Moreover, the decrease in 11βHSD2 enzyme activity may be related to several nutritional and environmental changes that occur during a lifetime. Henschkowski et al [31] reported that 11βHSD2 activity declines with age in hypertensive patients from 18 to 84 years and, Campino et al [32] show an inverse age-related concentration in serum cortisol and cortisone concentrations in healthy, normotensive subjects and this study shows that in a global population serum cortisone is associated with age (r = −0.3; *P* < .0001), but not cortisol or F/E. Similar to observations by Palermo et al [33], reduced 11βHSD2 activity correlates with decreased urinary excretion of free cortisone rather than increased urinary free cortisol excretion [28]. Thus, measuring cortisone seems to be the most appropriate assay of renal 11βHSD2 activity. Consequently, age-related 11βHSD2 impairment can partially account for the low-renin phenotype observed in elderly individuals. Recently, we demonstrated that NCAME has been identified in approximately 7% of the general population, and cortisone has been reported to be one of the best predictors of MR activation in subjects with NCAME. Thus, the simultaneous suppression of both renin and aldosterone entails a broad spectrum of disorders and requires a more complex evaluation, which should include the assay of cortisone levels.

We observed an association between cortisone and albuminuria (Table 4). This finding is similar to what occurs in PA, which could be associated with unregulated MR activation by cortisol that decreases with specific MR antagonism treatment [14]. Albuminuria has been considered as a marker of vascular dysfunction in both the kidney and systemic vasculature. Higher levels of albuminuria in patients with LRH indicate an impairment in glomerular permeability [34]. Previous studies have shown that normotensive children of hypertensive parents have increased urinary albumin excretion, which may further support the premise that microalbuminuria is an early finding in individuals with a predisposition to hypertension [35]. Moreover, urinary albumin excretion predicted BP progression in individuals without diabetes and without hypertension [36]. In this sense, it has been observed that MR antagonists reduce albuminuria, prevent cardiac fibrosis, and protect individuals with heart failure from progressive myocardial damage [37].

In this study, we observed that cortisone is inversely associated with PAI-1, which has been recognized as a biomarker of vascular risk, associated with activation of MR by active glucocorticoid. Previously, it has been reported that plasma PAI-1 levels have been shown to predict the risk of arterial hypertension [38, 39], to increase in normotensive subjects by activation of RAAS by either dietary sodium restriction or diuretic use, and decrease by interruption of RAAS with an angiotensin-converting enzyme inhibitor [40]. Another study showed in vitro that aldosterone acts synergistically with Ang-II to increase PAI-1 expression [41]. In 2004, Calo et al observed that higher concentrations of aldosterone were instead able to stimulate levels of PAI-1 in all subjects; this effect is probably mediated by MR activation [42]. In vascular tissue, PAI-1 promotes the accumulation of extracellular matrix and regulates vascular remodeling and perivascular fibrosis [43], leading to an imbalance in the fibrinolytic system that could lead to thrombotic events such as myocardial infarction or stroke [44].

## Conclusion

In summary, these findings highlight the concept that the low-renin phenotype is dictated not only by serum aldosterone levels but also by low cortisone, suggesting that a group of subjects with low renin activity could have a 11βHSD2 enzyme deficiency or a NCAME phenotype. Thus, low cortisone should be important and complementary in the screening of PA since a high aldosterone–renin ratio could identify false positive subjects for PA. Finally, our study also suggests that low cortisone levels could be associated with future incidence of vascular and kidney damage.

## Data Availability

Some or all datasets generated during and/or analyzed during the current study are not publicly available but are available from the corresponding author on reasonable request.

## Abbreviations

11βHSD2: 11β-hydroxysteroid dehydrogenase type 2
AME: apparent mineralocorticoid excess
AUC: area under the curve
BMI: body mass index
BP: blood pressure
DBP: diastolic BP
F/E: cortisol/cortisone
FEK: fractional excretion of potassium
LRH: low-renin hypertension
MR: mineralocorticoid receptor
NCAME: nonclassic AME
PA: primary aldosteronism
PAI: Plasminogen activator inhibitor
PRA: plasma renin activity
RAAS: renin–angiotensin–aldosterone system
ROC: receiver operating characteristic
SBP: systolic BP
